# Automated identification of keratinocyte cancers in pathology reports using large language models

**DOI:** 10.1371/journal.pdig.0001547

**Published:** 2026-07-09

**Authors:** Marloes Helder, Catherine M. Olsen, Nirmala Pandeya, Huanwei Wang, David C. Whiteman, Maciej Trzaskowski, Matthew H. Law

**Affiliations:** 1 Genetics and Skin Cancer, Population Health, QIMR Berghofer, Brisbane, Queensland, Australia; 2 School of Biomedical Sciences, Faculty of Health, Queensland University of Technology, Brisbane, Queensland, Australia; 3 Cancer Control, Population Health, QIMR Berghofer, Brisbane, Queensland, Australia; 4 Frazer Institute, Faculty of Health, Medicine and Behavioural Sciences, The University of Queensland, Brisbane, Queensland, Australia; 5 School of Public Health, University of Queensland, Brisbane, Queensland, Australia; 6 School of Public Health, Queensland University of Technology, Brisbane, Queensland, Australia; 7 Institute of Molecular Bioscience, University of Queensland, Brisbane, Queensland, Australia; 8 Genomics, Imaging, and AI Lab, Population Health, QIMR Berghofer, Brisbane, Queensland, Australia; 9 Co-Founder and Managing Director of Profenso, Brisbane, Queensland, Australia; 10 School of Biomedical Sciences, Faculty of Health, Medicine and Behavioural Sciences, University of Queensland, Brisbane, Queensland, Australia; The University of Sheffield, UNITED KINGDOM OF GREAT BRITAIN AND NORTHERN IRELAND

## Abstract

Keratinocyte cancers (KCs) are the most prevalent cancers in white-skinned individuals, yet remain underrepresented in cancer registries because reporting requirements differ greatly across jurisdictions. Manual extraction of KC subtypes from medical reports is labor-intensive and time-consuming, particularly as reports often document multiple co-excised skin lesions. Artificial intelligence offers automated solutions for disease phenotyping from unstructured clinical text. We fine-tuned the open-source large language model Meta-AI (LLaMA) 3.1-8B-Instruct on 26,179 manually reviewed pathology reports from 10,326 Australian QSkin Sun and health study participants who had histologically confirmed KCs. Independently validation was performed on 217 pathology reports from the Skin Tumors in Allograft Recipients cohort. The model achieved F1-scores above 0.90 for the four KC subtypes of interest: squamous cell carcinoma, basal cell carcinoma, keratoacanthoma, and intraepidermal carcinoma. Frequency-weighted mean F1-scores reached 0.84 (95% confidence interval: 0.843-0.846) for diagnosis classification and 0.83 (95% confidence interval: 0.826-0.828) for anatomical site. External validation demonstrated robust performance with F1-scores between 0.73-0.86 for the four KCs of interest. Our fine-tuned model QSkin-llama-3.1-8b works locally. It accurately classifies lesion counts, diagnoses, and anatomical sites. It processes 24 pathology reports per minute with minimal preprocessing, enabling scalable automated disease phenotyping for large health datasets.

## Author summary

We study keratinocyte cancers, the most common cancers in people with lighter skin. These cancers are not consistently recorded in cancer registries because they are so common and costly to document, which makes it harder to understand their true impact. Much of the relevant information exists in pathology reports, but these are written in complex language and often describe multiple skin lesions at once, making them slow and difficult to analyse manually.

In this work, we develop an automated approach to extract key information from these reports using artificial intelligence. We train a large language model on thousands of pathology reports and test how well it can identify different types of skin cancer and where they occur on the body. We find that the model performs with high accuracy and can process reports rapidly, analysing up to 24 reports per minute.

Our approach makes it possible to analyse large collections of medical data that would otherwise remain unused. This could improve how keratinocyte cancers are monitored and studied, and may help researchers and health systems better understand patterns of disease and plan care more effectively.

## Introduction

Keratinocyte cancers (KCs) are the most common cancers in predominantly white-skinned populations, with an estimated 69% of Australians having at least one excision for KC in their lifetime [1,2]. KCs account for the second-highest cancer-related costs in Australia, placing a substantial burden on the healthcare system, particularly among vulnerable groups like organ transplant recipients [3–5]. The two principal histological types of KCs are squamous cell carcinoma (SCC) and basal cell carcinoma (BCC), with related lesions including keratoacanthoma and intraepidermal carcinoma (IEC). The full disease burden of KCs remains unclear as these cancers are poorly captured in most cancer registries worldwide. This is largely due to the administrative burden of extracting data from the large volume of case reports, which are typically unstructured narrative documents [6,7]. Although many pathology departments worldwide now use standardised reporting checklists to ensure key elements such as clinical history, specimen type, and macroscopic and microscopic findings, reports still rely heavily on free-text entries [8]. Manual extraction and characterization of KCs and the specific histological subtype is labor-intensive and time-consuming. Moreover, > 50% of pathology reports document multiple skin lesions, resulting in a volume of information that limits its full utilization for epidemiological research [9].

Artificial intelligence and machine learning (ML) are reshaping complex disease phenotyping by enabling efficient analysis of unstructured data from large-scale biobanks and datasets [10,11]. Large language models (LLMs) are trained on extensive text data, enabling them to understand and generate human language. This capability allows them to identify patterns and relationships in unstructured clinical data, tasks that previously required specialized domain expertise.

Previous work by our group applied a supervised ML approach (referred to as the QSkin-ML model later in this paper) to identify KC cases and their histological subtypes from pathology records [12], achieving strong agreement with data extracted by human experts (κ = 0.8 - 0.9). However, this method relied on manually defined rules, such as excluding words that appeared in <10% or >90% of the reports, and required pre-processing of text into lesion-specific fragments when multiple lesions were documented. Because pathology reports lack a consistent and explicit structure, this fragmentation can limit the model’s ability to capture contextual relationships and long-range dependencies. As a result, it may overlook clinically relevant information such as rare terms, nuanced terminology, or subtle correlations in complex reports. For example, some reports combine multiple lesions in the summary section (i.e., “1-6, various locations, SCCs”), or include patient history that may suggest increased susceptibility to a particular type of skin cancer. Additionally, diagnosis type and anatomic site were modelled using separate algorithms, with performance sensitive to pre-processing choices. In contrast, LLMs offer deeper contextual understanding and flexibility. They can be fine-tuned for diverse tasks with minimal additional training and support multi-task learning, making them highly adaptable across applications [13].

Among available LLMs, encoder models directly analyze text and label individual words, while decoder models use task-specific instructions to generate structured outputs [14]. Decoder-only generative models such as LLaMA consistently outperform encoder-only models like BERT (Bidirectional Encoder Representations from Transformers) in diagnosis-related classification tasks [15,16]. While larger models generally yield better performance, they also demand significantly more computational resources and longer inference times [15,16]. Pretrained LLMs are typically trained on broad, general-purpose datasets, which provide extensive linguistic knowledge but limited domain specificity. Fine-tuning on specialized clinical datasets allow these models to adapt to domain-specific tasks [17]. Recent studies show that fine-tuned LLMs with 8 billion parameters outperform larger models, including GPT-4o and Mistral variants, in both accuracy and F1-score [18,19]. This makes fine-tuning a compelling strategy for balancing performance with resource efficiency.

Despite these methodological advances, ethical concerns related to privacy and the protection of sensitive health information remains a major barrier to further development of LLMs for extracting information from pathology records. This makes on-premise deployment in a secure computing environment essential. Most currently available LLMs are closed-source, meaning their internal architectures are not publicly accessible, and they can only be used via application programming interfaces. This requires medical records to leave local systems [11]. Among open-source LLMs with publicly available weights, LLaMA (Meta) and Falcon (TII) are two of the most widely adopted options [20]. LLaMA was specifically designed to support research applications. A previous study has shown that the 70B LLaMA outperforms the 180B Falson model across all standard benchmarks [20]. Our study uses the open-source LLaMA model that can be fine-tuned locally on data from the QSkin Sun and Health Study [21].

In this study, we aim to advance automated diagnostic classification in large-scale health datasets, enabling scalable and efficient extraction of diagnostic labels. We did this by using our fine-tuned LLM QSkin-llama-3.1-8b, derived from LLaMA-3.1-8B-Instruct. Comparison against our previous QSkin-ML evaluates not only potential gains in classification accuracy, but also gains in usability and workflow efficiency.

## Methods

### Ethics statement

All pathology data used in this analysis were derived from cohorts and collections that recruited participant with written informed consent. QSkin study ethical approval and oversight was provided by the QIMR Berghofer Human Research Ethics Committee (P1309, P2034, P3434). Further information about the QSkin cohort can be found in the cohort profile [21]. STAR cohort study participants provided written informed consent. Study protocols were approved by institutional and hospital Human Research Ethics Committees (HREC/12/QPAH/409; QIMR P1481). More information about the STAR cohort can be found in the cohort profile [4].

### Cohorts

This study leveraged data from the QSkin Sun and Health Study, a large Australian cohort established to investigate skin cancer development in Queensland, which has the world’s highest reported incidence. The cohort comprises 43,794 men and women aged 40–69 years, randomly sampled from Queensland in 2011. The dataset includes 26,179 pathology reports from 2011 to 2016, derived from 7 private pathology laboratories including Sullivan Nicolaides Pathology (16,578), Queensland Medical Laboratory (7,797), and IQ Pathology (965), each using bespoke formats. However, reports generally contained key elements such as clinical history, specimen type, macroscopic and microscopic findings. Each report contained one or more lesions. QSkin participants provided informed consent for the use of their data in research. Ethical approval and oversight were provided by the QIMR Berghofer Human Research Ethics Committee (P1309, P2034, P3434). Further information about the QSkin cohort can be found in the cohort profile [21].

For external validation, we used data from the STAR cohort, an observational study comprising transplant recipients (lung, kidney, liver) at high risk of skin cancer recruited from tertiary centers and diagnosed with histopathologically confirmed skin cancers [4]. The average age of this high-risk group was 54 years, recruited from the Princess Alexandra Hospital in Brisbane. The dataset includes 217 pathology reports from 2012 to 2016. All study participants provided written informed consent. Study protocols were approved by institutional and hospital Human Research Ethics Committees (HREC/12/QPAH/409; QIMR P1481). More information about the STAR cohort can be found in [4].

### Pathology report extraction

A gold standard dataset was previously created from QSkin pathology reports by either clinician-led manual classification of each lesion (n = 22,745) or using the QSkin-ML model (17,773) [12]. The reports were manually pre-processed to remove personal identifiers, as this information was not required for model training and validation. The extracted fields included lesion ID, diagnosis, anatomic site, and specific facial site. The lesion ID is a unique numeric identifier assigned to each lesion within a report. The diagnosis field captures the specific histopathological diagnosis for each lesion, encompassing both malignant and non-malignant entities. The anatomic site refers to the body location of the lesion, and for lesions located on the face, the specific facial subsite was recorded. The latter is clinically important as anatomical facial sites differ in their risk of tumor recurrence and metastasis, and play a key role in guiding surgical decision-making [22]. As the primary aim of this study is generating reliable counts and incidence estimates of keratinocyte cancers, we focused primarily on lesion count, diagnosis, and site.

**Fig 1:**
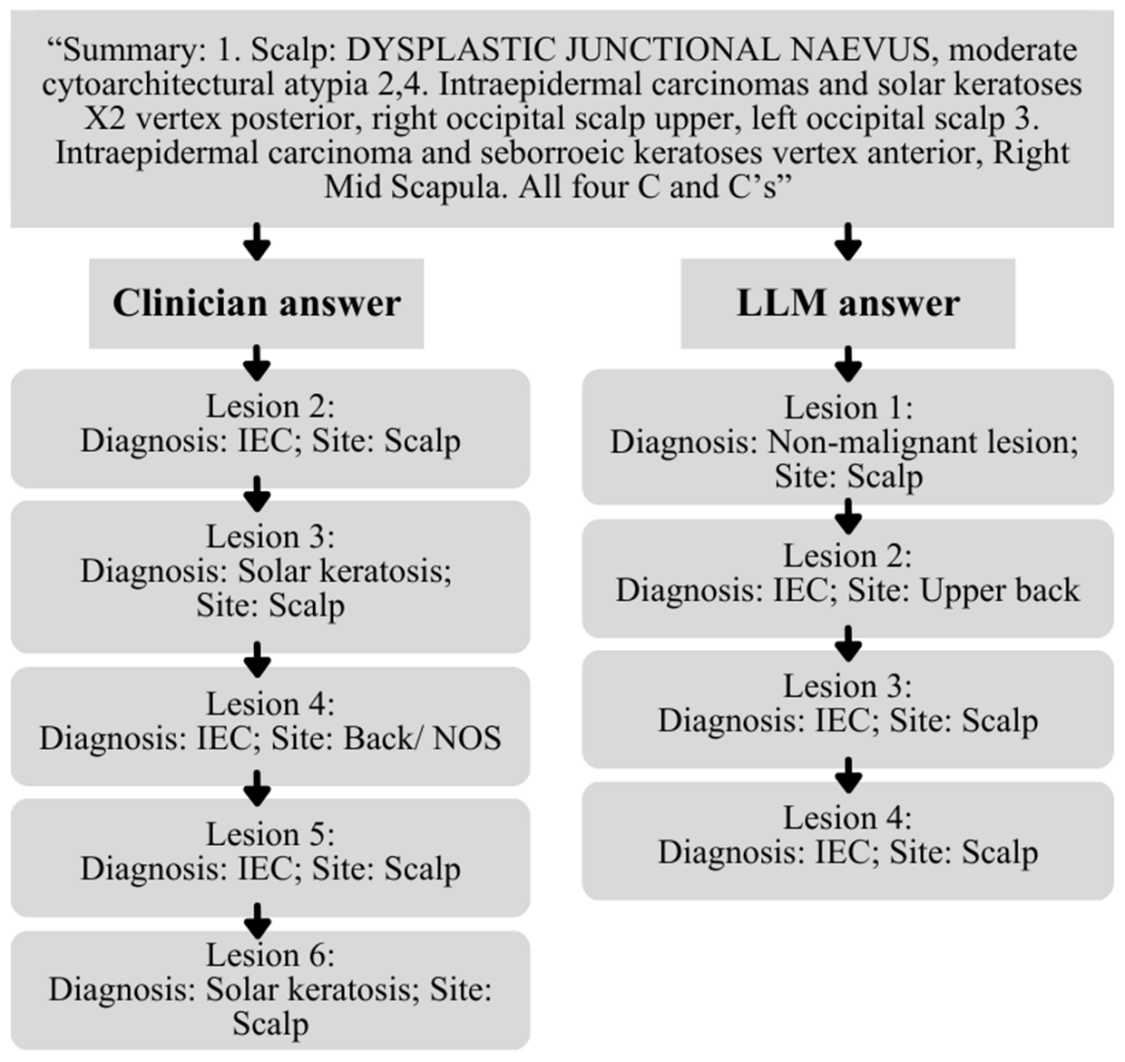
Example illustrating the differences in lesion counting between manual review and the large language model (LLM). Seborroeic keratoses and dysplastic neavus are benign lesions and were therefore not recorded in the manually reviewed dataset.

The manually classified dataset was originally developed to identify BCC, SCC, and keratoacanthoma for etiologic analyses [23]. Consequently, benign lesions were only extracted if they appeared before another invasive lesion later in the report, as this was necessary to preserve the sequential ordering of invasive lesions. Benign lesions appearing after the final invasive lesion were not assigned a lesion ID. For example, in a report describing four lesions in the order invasive, benign, invasive, benign, only the first three lesions would receive IDs, while the final benign lesion would be excluded. In contrast, the objective of the present study was to identify and enumerate all lesions. Accordingly, the fine-tuned QSkin-llama-3.1-8b extracts all lesions within a report, which can result in differences in lesion inclusion and numbering compared with the manually classified dataset (Fig 1).

Differences also arose in the handling of lesions with complex diagnoses. In the manually classified dataset, complex lesions (e.g., “an intraepidermal carcinoma arising in a solar keratosis”) were split into multiple entries, with each diagnosis assigned its own lesion ID. For example, a single lesion could be recorded as “1. IEC” and “2. solar keratosis”, and the following lesion would then receive ID 3. In contrast, QSkin-llama-3.1-8b assigns one primary diagnosis to each lesion (e.g., IEC), resulting in different representations of complex lesions (Fig 1).

Additional variation was observed in anatomical site classification. Some site categories in the manually classified dataset overlapped, such as *breast* and *upper chest/sternoclavicular*. Furthermore, pathology reports use varied terminology to describe lesion locations, which were not always labelled consistently during manual classification. Because QSkin-llama-3.1-8b is trained on these labels, this variability is reflected in the model outputs. As a result, direct comparison of raw anatomical site labels can produce apparent discrepancies even when the same location is identified. To address this, results are presented using both the original site labels and combined site categories. Specifically, *combined back* consists of *lower back*, *upper back*, and *back/NOS*. Combined hand consists of *back of hand* and *palmar skin, fingers*. *Combined upper chest* consists of *breast* and *upper chest/sternoclavicular*.

After matching the manual review outcomes with the available reports, 26,179 plain text reports describing 40,518 skin lesions were obtained (Table 1). Each record included lesion ID, primary diagnosis, anatomical site, and where applicable, specific facial location. The dataset was randomly split into training (80%, n = 20,943), validation (10%, n = 2,618), and testing (10%, n = 2,618) subsets. The validation set is used to tune model parameters and monitor performance during training, while the test set is held out entirely to evaluate the final model’s performance on unseen data.

**Table 1:** Counts of reports and lesions in the different datasets.

Dataset	N
Training set full reports	20,943
Number of separate lesions identified in the training set	40,519
Validation set full reports	2,618
Test set full reports	2,618
STAR cohort text full reports	57
STAR cohort text lesions	159
STAR cohort PDF full reports	160
STAR cohort PDF lesions	472

STAR cohort reports were first de-identified and two external test sets were created to evaluate QSkin-llama-3.1-8b generalizability. The first test set comprised 57 plain text reports documenting 159 lesions, comparable to the dataset used in the previous study. This dataset was manually classified by a medically trained staff member [12]. The second test set contained 160 scanned image PDFs, documenting 472 lesions. PDFs were converted to text using Python packages *pypfium2 (5.1.0)* and *pytesseract OCR (0.3.13)*. This dataset was manually reviewed independently by the first author (M.H.). Both test sets included lesion ID, primary diagnosis, anatomical site, and, where applicable, specific facial location.

### Model selection

The selection of the base model was guided by both computational feasibility and practical deployment considerations. Local high performance computing constraints restricted our evaluation to models with no more than 10 billion parameters and that support quantization; a model compression technique that reduces memory usage. Among open-source decoder models, LLaMA-3.1-8B-Instruct is one of the most widely adopted. Released in July 2024, it is open-access and free to download. Unlike Llama-3.1-8B, the Instruct version has undergone additional post-training to enhance its ability to follow instructions by using millions of human instructions and preference judgements to improve the pre-trained model [24]. LLMs convert text into tokens, which are the input units that an LLM can process. For each model call, there is a maximum number of tokens that can be processed at once, known as the context window. For LLaMA-3.1-8B-Instruct, this context window is 32,000 tokens per input instance, which is sufficient for all reports analyzed in this study. Because performance differences between models fine-tuned on the same dataset are generally small [18,25], we selected LLaMA-3.1-8B-Instruct for this study.

### Fine-tuning

We developed three model variants that predict lesion count, diagnosis, site, and when site is “Face” also the specific facial sites. The three models were: (1) a diagnosis-only model, predicting lesion count and diagnosis; (2) a site-only model predicting lesion count, lesion site, and facial site; and (3) a combined model predicting lesion count, diagnosis, lesion site, and facial site in a single output. We compared the performance of these models to determine whether separate models offer an improved accuracy over the single, multi-task model. All were trained using identical methodology, hyperparameters, and data, with differences only in the prompt focus and target outputs. Each model was instructed to return outputs strictly in JavaScript Object Notation (JSON) format, making it easier to integrate the results into analysis workflows.

To be able to fit the model on our Tesla V100 GPU with 32GB memory, we applied 4-bit quantization via the *BitsAndBytes* python library. In addition, we employed Parameter-Efficient Fine-Tuning (PEFT), which enables near full fine-tuning performance while significantly reducing computational demands [14]. Specifically, we used Low-Rank Adaption (LoRA) for the LLaMA-3.1-8B-Instruct model. LoRA operated by freezing the pre-trained model weights and injecting trainable low-rank decomposition matrices into each layer, allowing efficient adaptation without modifying the full parameter set [26]. Compared to retrieval-augmented generation (RAG) methods, PEFT has demonstrated superior accuracy when sufficient labelled data is available [27]. We adopted a zero-shot prompting approach, meaning we did not provide additional training examples in the prompt, as the model had already been trained on representative examples.

Model training followed a supervised learning framework, with data organized as structured question, context, and answer entries. Each question targeted specific lesion-level information, the context consisted of the full pathology report text, and the answer corresponded to the manual label.

Training parameters were selected to balance model performance with hardware constraints, following a setup comparable to that used by Saluja et al. and adjusted to our hardware infrastructure [18]. We used a LoRA rank of 8 and alpha of 16, based on empirical optimization for this task. A rank of 8 provides a favorable balance between parameter efficiency and task performance, and keeping computational costs low. The alpha of 16 ensures adequate adaptation to the domain-specific characteristics of the QSkin dataset, which differs substantially from the general training corpus used to pre-train LLaMA-3.1-8B-Instruct.

To expedite training and reduce memory usage, we applied a dropout rate of 0.05 and enabled gradient checkpointing. Fine-tuning was conducted over three epochs using a 4-bit quantized version of the model, reducing memory requirements to approximately 32 GB. The model was trained with a learning rate of 2e-5 using the adamw_bnb_8bit optimizer, which offers a balance between performance and memory efficiency. Together, these approaches make the training process feasible for resource-constrained environments, as it requires only a single consumer-level GPU [14]. Additional training parameters are available in the code.

### Prompt engineering

A uniform structure was used for all three models to ensure comparability. The only variation across models was the specified focus of the extraction. This is demonstrated using [].

User prompt combined model:

What is [Diagnosis, Site, and SiteFace] for each lesion from this text? Output it as a JSON object, just generate the JSON object without explanations.

System prompt combined model:

You are an expert pathology AI assistant. Your task is to extract all skin lesion information from the clinical text provided. Only assign [diagnoses and site] from the set of options below.

[Allowed diagnoses:

‘Squamous cell carcinoma (SCC),’ ‘scc re-excision - clear,’ ‘Basal cell carcinoma (BCC),’ ‘bcc re-excision - clear,’ ‘precursor lesions,’ ‘benign naevus,’ ‘Bowens disease,’ ‘Bowens disease giving rise to SCC,’ ‘Dysplastic naevus,’ ‘Intraepidermal carcinoma (IEC),’ ‘iec re-excision - clear,’ ‘Keratoacanthoma (KA),’ ‘Lentigo maligna,’ ‘Lentigo/solar lentigo,’ ‘Melanoma,’ ‘Non-malignant lesion,’ ‘No skin lesions,’ ‘Other,’ ‘Seborrhoeic keratosis,’ ‘Solar keratosis,’ ‘Squamo-proliferative lesions.’]

[Allowed sites

‘Abdomen,’ ‘Back/ NOS,’ ‘Back Of Hand,’ ‘Breast,’ ‘Buttock,’ ‘Ears,’ ‘Face,’ ‘Finger nail,’ ‘Forearm, Elbow, Wrist,’ ‘Hip,’ ‘Illegible,’ ‘Lower back,’ ‘Lower Leg, Ankle, Knee,’ ‘Neck,’ ‘Non-Skin,’ ‘No record,’ ‘Palmar Skin, Fingers,’ ‘Perineum,’ ‘Plantar Skin, Toes,’ ‘Scalp,’ ‘Shoulders,’ ‘Site,’ ‘Skin/NOS,’ ‘Thigh,’ ‘Top of feet,’ ‘Trunk/NOS,’ ‘UpperArm,’ ‘Upper back,’ ‘Upper chest/ Sternoclavicular.’]

[Allowed sites on the face:

‘Cheeks,’ ‘Chin/ Jaw,’ ‘Face/NOS,’ ‘Forehead,’ ‘Lips,’ ‘Nose,’ ‘Siteface,’ ‘Skin of orbit/ eyelid,’ ‘Temple.’]

Instructions:

1. Identify the [diagnosis and site] of ALL lesions mentioned in the text.

2. Assign a unique numeric ID to each lesion.

3. For each lesion, extract the following fields:

[- “Diagnosis”: primary diagnosis (must be from the allowed set)]

[- “Site”: primary site (must be from allowed sites)]

[- “SiteFace”: if site is ‘Face’, give specific location on face, otherwise leave as an empty string (must be from the allowed sites on the face)]

4. Output strictly as a valid JSON object with the following format:

[{"1":{"Diagnosis":"..","Site":"..","SiteFace":".."},"2":{"Diagnosis":"..","Site":"..","SiteFace":".."},..}]

5. Do NOT include explanations, extra text, or any formatting outside the JSON.

6. Make sure the JSON is syntactically correct and parsable.

### Performance evaluation and metrics

Performance was first assessed on the held-out QSkin test subset (n = 2,618), followed by evaluation on external STAR cohort data, including text reports (n = 58) and scanned PDF reports (n = 160). To measure variability in predictions, each evaluation was repeated five times on the same test set. Performance was assessed using standard Name Entity Recognition classification metrics, with manual classifications serving as the ground truth [28]. First, precision was measured as the proportion of correctly identified diagnoses among all diagnoses predicted by the model. Second, the accuracy was measured as the overall correctness of predictions (i.e., both positive and negative predictions), calculated as the ratio of true results to the total number of cases identified by the model. Recall was measured as the proportion of true positive cases correctly identified by the model. The F1-Score is the harmonic mean of precision and recall, providing a balanced measure of performance. Additionally, we calculated Cohen’s Kappa, which measures agreement between the model predictions and the gold standard classifications, adjusting for chance agreement. The formulas used were as follows where TP is true positives, FP is false positives, TN is true negatives, and FN is false negatives:


$$\begin{array}{c} precision\;=\;\frac{TP}{\left(TP+FP\right)} \end{array}$$

$$\begin{array}{c} accuracy\;=\;\frac{TP+TN}{\left(TP+TN+\;FP+FN\right)} \end{array}$$

$$\begin{array}{c} ecall\;=\;\frac{TP}{\left(TP+FN\right)} \end{array}$$

$$\begin{array}{c} F\text{1}\;=\;\frac{\left(\text{2}\times \;precision\;\times \;recall\right)}{\left(precision\;+\;recall\right)} \end{array}$$

$$\begin{array}{c} kappa\;=\;\frac{\text{2}\;\times \left(\;(\;TP\;\times TN\;)-(\;FN\;\times FP\;)\;\right)}{\left(TP\;+\;FP)\;\times \;(FP\;+\;TN)\;+\;(TP\;+\;FN)\;\times \;(\;FN+TN\;\right)}\; \end{array}$$

Model performance during training was monitored by tracking the training and evaluation loss. Training loss measures how closely the model’s predictions match the true labels in the training dataset at each stage of learning, while validation loss indicates how well the model performs on unseen data at each stage of training. A decreasing training loss curve indicates effective learning and improved predictive accuracy. Evaluations were conducted every 250 steps. In this study, we report the F1-score and Cohen’s kappa for LLM-predicted lesion outcomes compared with manually extracted outcomes. As LLM inference is stochastic, repeated runs can yield slightly different predictions; therefore, 95% confidence intervals are reported to quantify model variability on the same dataset. These results are compared with the performance of the QSkin-ML model [12].

### Lesion count evaluation

For the combined model, we assessed the accuracy of lesion ID assignment, where lesion ID refers to the sequential numbering of lesions within a report. LLM-derived lesion IDs were compared with manually assigned IDs in the QSkin test subset. This comparison was demonstrated using a confusion matrix, with manual lesion numbers in the rows and LLM-assigned numbers in the columns. To account for variability in the model’s outputs, the confusion matrix was aggregated across five independent runs. Agreement between model-derived and manually assigned lesion numbering was quantified using linearly weighted Cohen’s kappa with 95% confidence intervals. As noted above, lesion numbering differed slightly between the manual extraction and the LLM outputs, particularly for lesions with double diagnoses or benign lesions. To address this, we repeated the comparison in the STAR cohort, where lesion numbering was performed in the same manner as the LLM.

In addition, we conducted a sensitivity analysis focusing on lesion-level agreement independent of numbering. Agreement was defined by matching lesion diagnosis, anatomic site, and facial subsite within each report. We used the datasets in which related sites were combined for back, hand, and upper chest. Across the full test set, we calculated overall precision, defined as the proportion of predicted lesions that corresponded to manually extracted lesions, aggregated across all reports.

## Results

**Fig 2:**
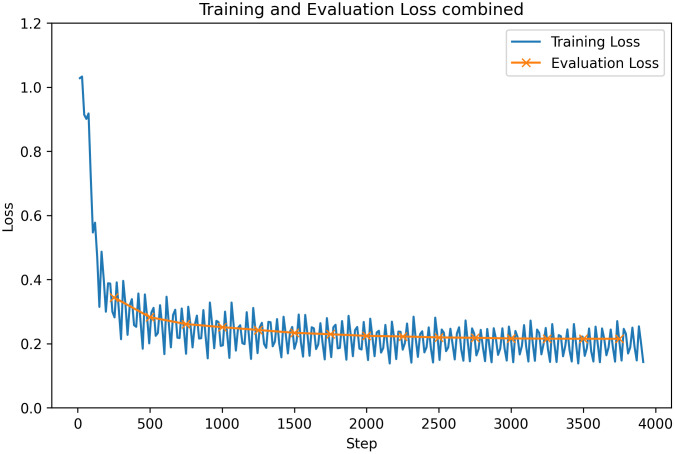
Training and evaluation loss fine-tuning combined model. Epoch: 3; leaning-rate: 2e-5; logging steps: 15; evaluation steps: 250.

To evaluate how well the LLaMA model was learning across diagnosis, lesion site, and combined classification tasks, we first examined the training and validation loss curves (Fig 2 and S1 Fig). Training was conducted using 80% of the dataset (n = 20,943), while validation was performed on a further 10% (n = 2,618). The last 10% was held out to evaluate the model’s final performance (Table 1). The longest report in our dataset contained 4,317 tokens, which is far below the maximum context window of the model (32,000 tokens per record). This indicates that input length for each discrete record is not a practical limitation for our model.

### Training loss

All three fine-tuned models follow a similar flow in the training and validation loss, therefore, only the combined model is presented in Fig 2. Results for the two separate models can be found in the Supplements (S1 Fig). Training loss starts at approximately 1.0, with the most substantial decrease occurring within the first 250 steps, indicating rapid initial learning. Following this, the loss gradually stabilizes around 0.2, with minor oscillations that reflect normal variation rather than instability. The valuation loss smoothly decreases to approximately 0.2, mirroring the training loss trend. The validation loss remained nearly parallel to the training loss, suggesting that the model did not overfit and was generalizing well to unseen data. These patterns indicate that the model effectively converged on the training dataset and achieved stable learning dynamics.

### Comparison of the three models

The mean F1-score for each diagnosis and site type was calculated by weighing the frequency of each subtype in the training dataset. The combined model was evaluated separately for diagnosis and site, with site including face-specific locations. For diagnosis classification, the combined model scored 0.844 (95% CI [0.843, 0.846]), compared to 0.822 (95% CI [0.820, 0.824]) for the diagnosis-specific model. Similarly, for site classification, the combined model achieved a weighted mean F1-score of 0.827 (95% CI [0.826, 0.828]), which was only marginally higher than the score from the site-specific model (0.824, 95% CI [0.823, 0.825]). In addition to the modest improvement in performance, using a combined model is more convenient because it simplifies deployment, reduces computational resources, and avoids maintaining multiple models. For these reasons, we proceeded with the fine-tuned combined model (referred to as QSkin-llama-3.1-8b in this paper).

### Performance metrics

The F1-scores and Cohen’s Kappa statistics, along with their 95% confidence intervals for QSkin-llama-3.1-8b are presented in Table 2 (diagnosis) and Table 3 (lesion site). The frequency (N) reported in the table indicates how often each diagnosis and site appeared in the manual classified dataset. F1-scores and Cohen’s Kappa were calculated using predictions on the test set. To ensure reliable performance estimates, only diagnoses occurring at least 10 times in the test set are included. Fig 3 illustrates the relationship between log F1-score and the frequency of each lesion diagnosis (Fig 3A) and site (Fig 3B).

**Table 2:** F1-score and Cohen’s kappa for each lesion diagnosis obtained from QSkin-llama-3.1-8b. The 95% confidence intervals reflect inter-run variability across five independent model executions. For the diagnosis only model see S1 Table in the Supplements. N is the frequency of each diagnosis in the training dataset.

Diagnosis	F1-score	Kappa	N training
BCC	0.95 [0.95, 0.95]	0.93 [0.93, 0.93]	8,881
melanoma re-excision - clear	0.88 [0.85, 0.90]	0.87 [0.85, 0.90]	413
melanoma*	0.75 [0.72, 0.78]	0.75 [0.72, 0.77]	498
intraepidermal carcinoma (IEC)	0.90 [0.89, 0.90]	0.87 [0.87, 0.88]	6,085
SCC	0.91 [0.90, 0.91]	0.90 [0.89, 0.90]	3,107
keratoacanthoma	0.95 [0.95, 0.96]	0.95 [0.94, 0.95]	790
dysplastic naevus**	0.19 [0.09, 0.30]	0.19 [0.09, 0.30]	139
BCC re-excision - clear	0.72 [0.70, 0.74]	0.72 [0.70, 0.74]	455
solar keratosis**	0.76 [0.75, 0.77]	0.73 [0.72, 0.73]	4,604
Other	0.03 [0.0, 0.06]	0.02 [0.0, 0.06]	325
seborrhoeic keratosis**	0.16 [0.13, 0.19]	0.16 [0.12, 0.19]	233
SCC re-excision - clear	0.66 [0.61, 0.71]	0.66 [0.61, 0.71]	241
lentigo maligna*	0.29 [0.21, 0.38]	0.29 [0.20, 0.38]	163
squamo-proliferative lesions	0.43 [0.39, 0.47]	0.43 [0.39, 0.46]	158
IEC re-excision - clear	0.55 [0.46, 0.64]	0.55 [0.46, 0.64]	178
non-malignant lesion**	0.84 [0.83, 0.84]	0.80 [0.80, 0.80]	5,654
Combined melanoma*	0.87 [0.86, 0.87]	0.86 [0.85, 0.87]	–

Excluded diagnosis with an occurrence of ≤ 10 in the test set: *benign naevus*, *lentigo/solar lentigo*, *IEC giving rise to SCC* and *no skin lesions*.

*In the manual classification set, *lentigo maligna* was sometimes recorded as *melanoma* and at other times as *lentigo maligna*, which reduced concordance between classifications. Therefore, a combined melanoma category was created in which *lentigo maligna* was recorded as *melanoma*. Merging happened after training, therefore, no frequency in training set is given.

**The model classifies all types of benign lesions as the general category *non-malignant lesion*, instead of specifying the subclasses *seborrhoeic keratosis*, *dysplastic naevus, benign naevus, lentigo/solar lentigo*, or *solar keratosis*.

**Table 3:** F1-score and Kappa including 95% confidence interval for each lesion site from QSkin-llama-3.1-8b. The 95% confidence intervals reflect inter-run variability across five independent model executions. When the lesion site is “face”, the specific location on the face is also extracted. For the site only model, see S2 Table in the Supplements. N is the frequency of each site in the training dataset.

Site	F1-score	Kappa	N training
Abdomen	0.88 [0.81, 0.94]	0.87 [0.81, 0.94]	220
Back of hand*	0.86 [0.85, 0.86]	0.85 [0.84, 0.86]	1,458
Back/NOS*	0.60 [0.59, 0.62]	0.59 [0.58, 0.61]	837
Breast*	0.29 [0.27, 0.32]	0.29 [0.26, 0.32]	346
Ears	0.89 [0.89, 0.90]	0.89 [0.89, 0.90]	1,038
Forearm, elbow, wrist	0.86 [0.86, 0.86]	0.84 [0.84, 0.85]	3,244
Lower back*	0.52 [0.49, 0.56]	0.51 [0.48, 0.55]	644
Lower leg, ankle, knee	0.92 [0.91, 0.92]	0.91 [0.90, 0.91]	4,170
Neck	0.91 [0.91, 0.91]	0.91 [0.90, 0.91]	1,496
Non-skin	0.89 [0.88, 0.90]	0.88 [0.87, 0.89]	1,410
Palmar skin, fingers*	0.60 [0.58, 0.63]	0.60 [0.58, 0.63]	374
Scalp	0.81 [0.80, 0.82]	0.81 [0.80, 0.82]	812
Shoulders	0.81 [0.81, 0.81]	0.80 [0.79, 0.80]	1,792
Thigh	0.89 [0.89, 0.90]	0.89 [0.88, 0.90]	844
Top of feet	0.87 [0.85, 0.88]	0.87 [0.85, 0.88]	227
Upper back*	0.67 [0.66, 0.68]	0.65 [0.64, 0.66]	1,636
Upper chest/sternoclavicular*	0.72 [0.71, 0.73]	0.71 [0.70, 0.72]	1,428
Upper arm	0.86 [0.85, 0.87]	0.85 [0.84, 0.86]	1,612
Combined back*	0.81 [0.80, 0.82]	0.79 [0.78, 0.78]	–
Combined hand*	0.86 [0.85, 0.86]	0.85 [0.84, 0.86]	–
Combined upper chest*	0.83 [0.83, 0.84]	0.82 [0.82, 0.83]	–
Face	0.90 [0.90, 0.90]	0.87 [0.87, 0.87]	7,823
Cheeks	0.76 [0.75, 0.77]	0.74 [0.73, 0.75]	1,853
Chin/jaw	0.71 [0.71, 0.72]	0.71 [0.70, 0.72]	458
Forehead	0.81 [0.79, 0.82]	0.80 [0.78, 0.81]	1,301
Lips	0.86 [0.85, 0.87]	0.86 [0.85, 0.87]	467
Nose	0.89 [0.88, 0.89]	0.88 [0.88, 0.89]	1,850
Skin of orbit/eyelid	0.69 [0.69, 0.70]	0.69 [0.68, 0.69]	776
Temple	0.74 [0.72, 0.76]	0.74 [0.71, 0.76]	833

Excluded site with an occurrence of ≤ 10 in the test set: *buttock*, *hip*, and *plantar skin, toes*.

*Overlapping sites which are combined into common anatomical regions. *Combined back* consists of *lower back*, *upper back*, and *back/NOS*. Combined hand consists of *back of hand* and *palmar skin, fingers*. *Combined upper chest* consists of *breast* and *upper chest/sternoclavicular*. Merging happened after training, therefore, no frequency in the training set is provided.

### Comparison QSkin-llama-3.1-8b with QSkin-ML model

**Fig 3:**
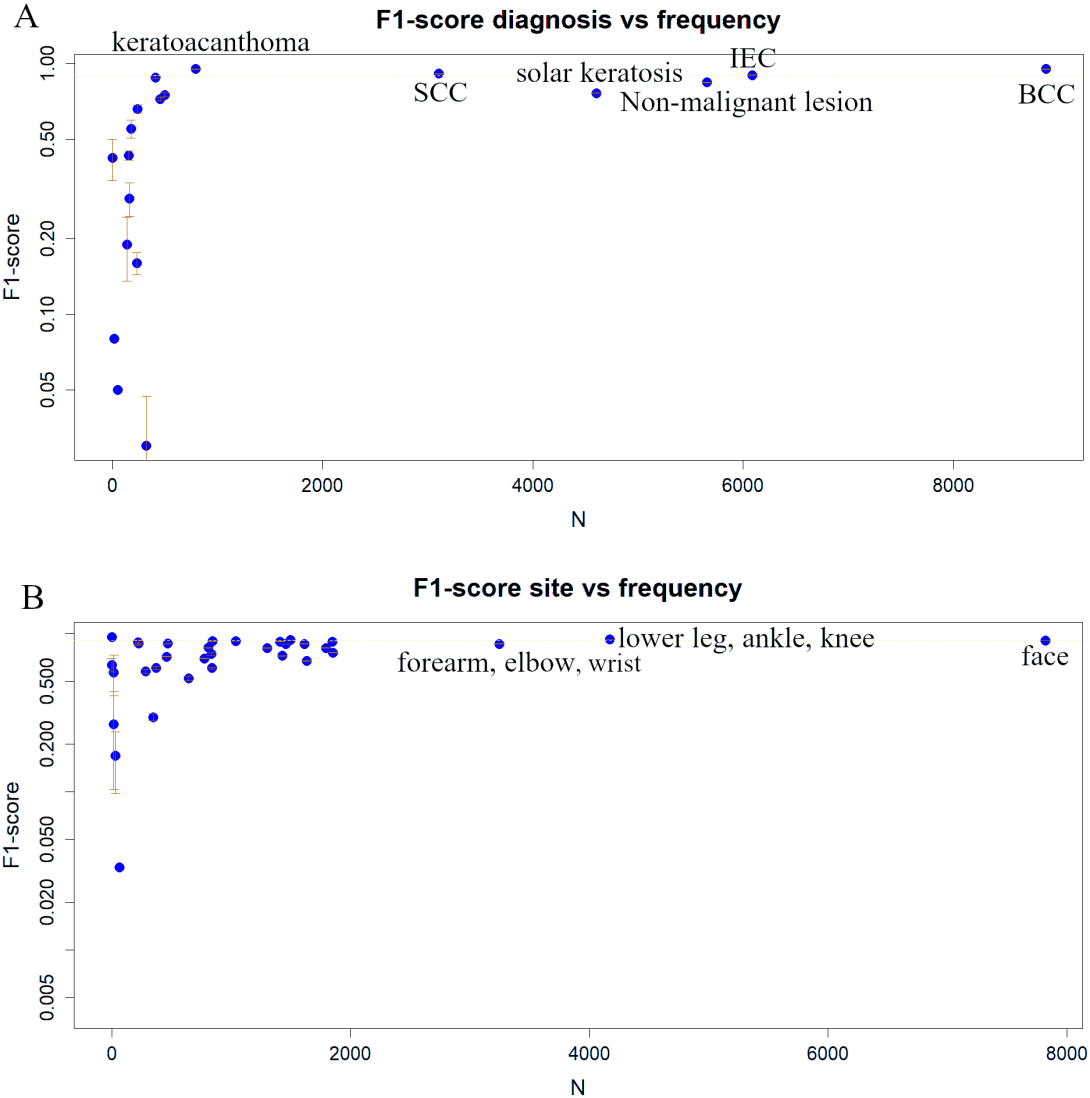
Log_10_ F1-score of test set compared to frequency of the diagnosis or site in the training dataset for QSkin-llama-3.1-8b. Y-axis is the log_10_ F1-score ranging from 0 to 1, with 1 being a perfect match. X-axis is the number of occurrences of the diagnosis in the training dataset. Blue dots indicate different skin cancer diagnoses or sites, grey error bars the 95% confidence intervals, red dotted line indicate a F1-score of 0.90. (A) Diagnosis. (B) Lesion site.

### Lesion count

Agreement between lesion counts derived from QSkin-llama-3.1-8b and those obtained manually was substantial, with a linear weighted κ of 0.798 (95% CI [0.796, 0.799]). Only a few extreme discrepancies were observed, primarily due to the manual classification set excluding most benign lesions (here malignant lesions were prioritized and only particular benign lesions were recorded). The model produced spurious outputs (hallucinations) in five reports across the five combined prediction runs, each comprising a full pass through the 2,618 reports in the test set (0.04%). These hallucinations were removed by retaining only the structured JSON component of the LLM output. Agreement between lesion counts derived from QSkin-llama-3.1-8b and those from the external STAR cohort yielded a linear weighted κ of 0.942 (95% CI [0.923, 0.962]). This indicates high concordance and demonstrates that the model accurately predicts lesion counts in a cohort with similar defined lesion numbering (S3 Table).

In addition, we performed a sensitivity analysis where we evaluated lesion-level agreement by comparing each predicted lesion to the manually reviewed lesions within the same report, considering a match if the diagnosis, site, and site face all exactly corresponded. Total precision was then calculated as the proportion of predicted lesions that had a corresponding match in the manual classification. Across the five model runs, we observed a mean precision of 0.748 (95% CI [0.742, 0.754]), indicating that 75% of predicted lesions corresponded to a manually reported lesion with identical attributes. The remaining 25% of predictions reflected discrepancies in diagnosis, site, or site face assignment.

### External validation metrics

The F1-scores and Cohen’s Kappa statistics, along with their 95% confidence intervals for the external STAR text and PDF test sets are presented in Table 4 (diagnosis) and Table 5 (lesion site). The frequency (N) indicates how often each diagnosis and site appeared in the STAR datasets. In the first STAR cohort test set, 5 diagnoses were represented with more than 10 occurrences, whereas the second STAR cohort test set included 8 diagnoses meeting this threshold. The model classified 9 out of 10 of the *benign naevus* in the PDF test set as *non-malignant lesion.* Both the manual classification set and the model showed inconsistent use of *Forearm, elbow, wrist* and *Upper arm* in the text test set*.*

**Table 4:** F1-score and Kappa including 95% confidence interval for each lesion diagnosis from the combined model in the two STAR cohort validation sets (text and PDF pathology reports). N is the frequency of each diagnosis in the test dataset.

Diagnosis	F1-score text	Kappa text	N text
BCC	0.86 [0.84, 0.88]	0.84 [0.82, 0.86]	16
intraepidermal carcinoma (IEC)	0.78 [0.77, 0.78]	0.60 [0.59, 0.61]	28
SCC	0.73 [0.71, 0.75]	0.70 [0.68, 0.72]	20
solar keratosis*	0.55 [0.53, 0.57]	0.48 [0.46, 0.51]	12
non-malignant lesion*	0.66 [0.60, 0.71]	0.60 [0.54, 0.66]	14
**Diagnosis**	**F1-score PDF**	**Kappa PDF**	**N PDF**
BCC	0.82 [0.80, 0.84]	0.77 [0.74, 0.79]	95
intraepidermal carcinoma (IEC)	0.81 [0.80, 0.82]	0.71 [0.70, 0.73]	166
SCC	0.76 [0.74, 0.78]	0.72 [0.69, 0.74]	71
solar keratosis*	0.56 [0.51, 0.61]	0.51 [0.46, 0.56]	58
Benign naevus*	0	0	10
Non-malignant lesion*	0.28 [0.23, 0.33]	0.21 [0.16, 0.27]	27
Other	0	0	11
Seborrhoeic keratosis*	0.16 [0.10, 0.22]	0.16 [0.09, 0.22]	20

Excluded diagnoses with an occurrence of ≤ 10 in the text test set: *keratoacanthoma*, *IEC re-excision - clear*. Excluded diagnoses in the PDF test set: *keratoacanthoma*, *benign naevus*, *dysplastic naevus*, *melanoma*, *no skin lesions*, *solar lentigo*, and *squamo-proliferative lesions*.

*The model classifies all types of benign lesions as the general category *non-malignant lesion*, instead of specifying the subclasses *seborrhoeic keratosis*, *dysplastic naevus, benign naevus, lentigo/solar lentigo*, or *solar keratosis*.

**Table 5:** F1-score and Kappa including 95% confidence interval for each lesion site from the combined model in the two STAR cohort test sets (text and PDF pathology reports). When the lesion site is “face”, the specific location on the face is also extracted. N is the frequency of each site in the test dataset.

Site	F1-score text	Kappa text	N text
Forearm, elbow, wrist	0	0	11
Upper chest/sternoclavicular*	0.77 [0.70, 0.83]	0.73 [0.65, 0.80]	13
Combined back*	0.69 [0.64, 0.74]	0.66 [0.61, 0.72]	10
Combined upper chest*	0.87 [0.85, 0.89]	0.84 [0.82, 0.87]	15
Face	0.86 [0.85, 0.87]	0.83 [0.82, 0.84]	57
**Site**	**F1-score PDF**	**Kappa PDF**	**N PDF**
Back of hand*	0.70 [0.66, 0.74]	0.69 [0.64, 0.74]	17
Back/ NOS*	0.38 [0.30, 0.47]	0.37 [0.28, 0.46]	14
Ears	0.85 [0.81, 0.89]	0.84 [0.80, 0.88]	30
Forearm, elbow, wrist	0.83 [0.79, 0.86]	0.79 [0.75, 0.83]	77
Lower leg, ankle, knee	0.81 [0.78, 0.85]	0.79 [0.75, 0.82]	71
Neck	0.77 [0.75, 0.79]	0.76 [0.74, 0.78]	20
Scalp	0.84 [0.79, 0.88]	0.83 [0.79, 0.88]	13
Shoulders	0.81 [0.77, 0.84]	0.79 [0.76, 0.83]	36
Thigh	0.90 [0.85, 0.94]	0.89 [0.85, 0.94]	11
Upper chest/sternoclavicular*	0.61 [0.47, 0.76]	0.60 [0.45, 0.75]	16
Upper arm	0.70 [0.59, 0.81]	0.69 [0.57, 0.80]	15
Combined back*	0.57 [0.55, 0.60]	0.56 [0.53, 0.58]	17
Combined hand*	0.76 [0.71, 0.80]	0.75 [0.70, 0.79]	24
Combined upper chest*	0.66 [0.54, 0.79]	0.65 [0.52, 0.78]	14
Face	0.88 [0.85, 0.91]	0.83 [0.80, 0.87]	130
Cheeks	0.57 [0.54, 0.60]	0.55 [0.52, 0.58]	26
Chin/jaw	0.67 [0.63, 0.70]	0.65 [0.61, 0.69]	14
Forehead	0.65 [0.56, 0.74]	0.63 [0.53, 0.72]	20
Nose	0.84 [0.82, 0.86]	0.83 [0.81, 0.86]	26
Skin of orbit/eyelid	0.26 [0.15, 0.37]	0.26 [0.15, 0.37]	12
Temple	0.68 [0.62, 0.73]	0.66 [0.60, 0.72]	21

Excluded sites with an occurrence of ≤ 10 in the text test set: *back of hand*, *back/ NOS*, *breast, ears, lower back, “lower leg, ankle, knee”, neck, non-skin, “palmar skin, fingers”, scalp, shoulders, thigh, top of feet, upper back, upper arm, cheeks, chin/jaw, forehead, lips, nose,* and *skin of orbit/eyelid*. Excluded sites with an occurrence of ≤ 10 in the PDF test set: *abdomen, breast, non-skin, “palmar skin”, fingers, top of feet, upper back,* and *lips*.

*Overlapping sites which are combined into common anatomical regions. *Combined back* consists of *lower back*, *upper back*, and *back/NOS*. Combined hand consists of *back of hand* and *palmar skin, fingers*. *Combined upper chest* consists of *breast* and *upper chest/sternoclavicular*.

## Discussion

Keratinocyte cancers represent the most common cancers in white-skinned populations, yet their true disease burden remains difficult to quantify due to differences in reporting practices and the unstructured nature of pathology reports. Manual extraction of KC diagnoses from medical reports is labor-intensive. Thus, although KCs result in substantial morbidity and treatment costs, reliable and up-to-date incidence statistics for these cancers are lacking [7]. Thompson et al. previously addressed this challenge using a supervised learning approach to classify KC related pathology reports [12]. In the present study, we extend this work by developing QSkin-llama-3.1-8b, a fine-tuned large language model designed to extract skin cancer diagnosis, lesion count, and lesion site information from free-text pathology reports containing one or multiple lesions. We compared our model with QSkin-ML model in terms of ease of use, scalability, and performance.

Our findings demonstrate that an 8 billion parameter model (LLaMA-3.1-8B-Instruct), sourced locally and trained on a labeled dataset, can achieve performance comparable to manual extraction by subject-matter experts (Tables 2 and 3). QSkin-llama-3.1-8b demonstrated particularly high accuracy for the four KC diagnoses of interest (SCC, BCC, keratoacanthoma, and IEC), with performance generally improving as the diagnosis and site frequency in the training dataset increased. QSkin-llama-3.1-8b also demonstrated robust performance across the complex external STAR test sets, achieving F1-scores between 73% and 86% for SCC, BCC, and IEC, with comparable results between text and the scanned PDF datasets. Discrepancies in performance for specific diagnoses or lesion sites between the two external test sets may reflect inter-rater variability, as different researchers manually reviewed each STAR dataset. However, it should be noted that this external dataset is relatively small and only includes the main skin cancer diagnoses and anatomical sites. Compared to the QSkin-ML model, QSkin-llama-3.1-8b showed improved F1-scores for three of the four KC diagnoses of interest, with both models scoring similar results for SCC (Table 6). For the other diagnoses, QSkin-llama-3.1-8b did improve for SCC re-excision, but performed less well compared to the QSkin-ML model for melanoma, BCC re-excision, squamo-proliferative lesions, and non-malignant lesions (S4 Table). This is due to QSkin-llama-3.1-8b prioritizing melanoma and non-malignant lesions over the specific subclasses. For lesion location, the QSkin-ML model showed an overall slightly higher F1-score compared to QSkin-llama-3.1-8b. However, when considering the combined site categories (back, hand, and upper chest), performance was comparable between the two models (S5 Table). Lesion ID and site of lesion were not collected in the external STAR cohort validation set in the Thompson et al. study, therefore, F1-scores per diagnosis and site could not be compared for this cohort [12].

**Table 6:** F1-score for the four KC diagnoses of interest predicted by QSkin-llama-3.1-8b (LLM) and the QSkin-ML (ML) model. *95% confidence intervals reflect variability across repeated LLM runs.

Diagnosis	F1-score LLM*	F1-score ML
BCC	0.95 [0.95, 0.95]	0.93
SCC	0.91 [0.90, 0.91]	0.91
Keratoacanthoma	0.95 [0.95, 0.96]	0.89
IEC	0.90 [0.89, 0.90]	0.86

When focusing on ease of use and scalability, QSkin-llama-3.1-8b can be directly applied to raw text. It effectively handles non-standard character encodings (e.g., Ã¢Â€ ™˜) arising from text extraction, and successfully processed all reports. In contrast, QSkin-ML model relied on manual feature engineering and fixed text representations, requiring adaptation of preprocessing rules to suit different text formats [12]. Approximately 7% of reports in the second validation set of QSkin-ML model could not be processed due to formatting irregularities, a limitation not observed with our model. Another aspect is that the QSkin-ML model encountered difficulties when separating 8 different BCC lesions within a single pathology report, and the lower accuracy in the external STAR cohort was said to be attributed to the size of the pathology reports; which frequently describe over 10 lesions in a single report. QSkin-llama-3.1-8b does not require explicit lesion text separation, and appears capable to perform well above 10 lesions (Table 7 and S3 Table). In five reports, the model produced hallucinated outputs in which it became trapped in a repetitive text-generation loop. These responses were truncated when reaching the set maximum token limit and therefore failed to produce a complete JSON structure. This issue was then resolved by retaining only the valid JSON content from the output.

**Table 7:** Lesion count for each pathology report for the QSkin-llama-3.1-8b answer and the manual classification.

	Lesion count by QSkin-llama-3.1-8b																			
Lesion count by manual classified dataset	NaN**	1	2	3	4	5	6	7	8	9		10		11		12		13	15	18
1	3	8259	475	80	11	2	5	5	0	0		0		0		0		0	4*	1*
2	2	456	1960	117	5	15	0	0	0	0		0		0		0		5*	0	0
3	9	21	209	680	51	15	0	0	0	0		0		0		0		0	0	0
4	1	0	26	65	213	10	10	0	0	0		0		0		0		0	0	0
5	0	6	7	17	19	90	11	0	0	0		0		0		0		0	0	0
6	0	5	5	5	5	20	41	14	0	0		5		0		0		0	0	0
7	0	0	0	0	10	10	18	17	0	0		0		0		0		0	0	0
8	0	0	0	0	0	0	15	10	10	0		0		0		0		0	0	0
9	0	0	0	0	0	0	5	0	0	5		0		0		0		0	0	0
10	0	0	0	0	0	0	0	0	0	5		10		0		0		0	0	0
11	0	0	0	0	0	0	0	0	0	0		0		2		8		0	0	0
12	0	0	0	0	0	0	0	0	0	0		0		0		0		0	0	0
13	0	0	0	0	0	0	0	0	0	0		0		0		0		0	0	0
15	0	0	0	0	0	0	0	0	0	0		0		0		0		0	0	0
18	0	0	0	0	0	0	0	0	0	0		0		0		0		0	0	0

*Two reports showed extreme discrepancies between model outputs and manual counts due to exclusion of benign lesions in manual classification. One report included reports on 13 separate lesions, but only two malignant lesions were manually recorded in the database. The second report included 15 lesions with manual reporting available for only one malignant lesion.

**NaN means the LLM did not give an output for this lesion number.

Agreement between lesion counts derived by QSkin-llama-3.1-8b and the manual lesion count was substantial (linear weighted κ 0.798, 95% CI [0.796, 0.799]). However, agreement alone does not indicate whether QSkin-llama-3.1-8b extracts all lesions, as the manual lesion count excluded non-relevant lesions and double-counted cases where a secondary diagnosis was recorded. In the external cohort, where lesion counting followed an approach more closely aligned with the model, agreement was substantially higher (linear weighted κ 0.942, 95% CI [0.923, 0.962]). The sensitivity analysis that evaluates agreement based on the matching of diagnosis, site, and site face yielded a mean precision of 0.748 (95% CI [0.742, 0.754]). This indicates that 75% of predicted lesions corresponded exactly to a manually reported lesion. The remaining 25% of predictions results from mismatches in either diagnosis, site, or site face.

After reviewing mismatches in lesion diagnosis and site, we found that roughly half of the model’s predictions were correct, suggesting that the manual classification set may not represent a perfect gold standard and may itself contain inconsistencies. Because the model is trained on human-labelled data, its performance is inherently limited by the quality of the training set. One limitation that may arise from this is the tendency of QSkin-llama-3.1-8b to prioritize the general category *non-malignant lesion* over specific subclasses, such as *seborrhoeic keratosis*, *dysplastic naevus, benign naevus, lentigo/solar lentigo*, and *solar keratosis*. Similarly, *lentigo maligna,* a distinct melanoma subtype, is often categorized as *melanoma*. While these classifications are technically correct, they reduce diagnostic granularity. Consequently, the model is less suitable for research focused on these subtypes. This pattern is consistent with findings reported by Saluja et al., whose fine-tuned model also showed difficulty distinguishing between closely related or anatomically overlapping classes [18]. When we attempted to incorporate subclass information into the prompt, overall model performance on the other instructions declined. For example, the model no longer consistently selected from the predefined diagnosis and site categories and instead generated free-text responses such as “Solar keratosis with lichen simplex chronicus” or “Bridge of nose”. Therefore, the original prompt was retained. During model evaluation, this noise was addressed by combining broader diagnostic categories and overlapping anatomical sites into unified categories after the model had processed the reports.

A key strength of this work is the demonstration that resource-efficient fine-tuning of an open-source 8B parameter model can achieve strong performance across tens of thousands of pathology reports. Both training and deployment can be performed using a consumer-level GPU with 32GB GPU memory (of which approximately 80% was used), a single CPU, and 6.35 GB RAM. These resources are available in many hospital and research settings, supporting the practical feasibility of implementing this approach without large-scale computational infrastructure. The total training time, which only needs to be performed once, was 14.5 hours of CPU time. S2 Fig shows GPU and GPU memory usage during training. During deployment, the fine-tuned model processed approximately 24 reports per minute, corresponding to an average of 2.5 seconds per report. Processing the entire test set (n = 2,618) required 1 hour and 50 minutes in total. In addition, unlike many BERT-based encoder models limited to 512 input tokens [15], QSkin-llama-3.1-8b can process substantially longer inputs up to 32,000 tokens, making it suitable for lengthy multi-lesion reports. Importantly, our dataset is representative of worldwide clinical practice as it is based on the standardized checklist, yet remains reliant on free-text narrative inputs, underlining its potential for broader deployment beyond the current setting.

Future improvements could include additional fine-tuning on rare cases with an increased training set, that matches the size of the well performing types (>2,000 lesions). One potential approach would be to augment rare classes with high-quality synthetic training examples generated by larger flagship models. Furthermore, future exploration of prompt engineering and optimization of LoRA hyperparameters may lead to performance improvements while maintaining the same model size and training dataset. Due to resource constraints, opportunities to rerun and refine model training were limited; therefore, the prompts and parameter settings were largely based on examples reported in the literature. While our model performed well, the selected configuration may not represent the most optimal setup for this task. With the growing adaptation of multimodal LLMs, a potential enhancement would be to include additional data modalities when available, such as lesion images or supplementary pathology details. To enhance generalizability across laboratories, a retrieval-augmented generation (RAG) framework could be integrated, providing examples of local structures without retraining the model [28].

In conclusion, fine-tuning a relatively small, open-source LLM offers an efficient, accurate, and privacy-preserving solution for extracting lesion count, skin cancer diagnoses, and lesion sites from unstructured pathology reports. QSkin-llama-3.1-8b requires minimal preprocessing to accommodate diverse formats, only a single standardized conversion step is necessary before image PDFs can be processed by the model. Although F1-scores on the external STAR datasets were slightly lower than those on the QSkin test set, the results nonetheless demonstrate the model’s ability to generalize to previously unseen pathology reports across diverse formats. In addition, the model generates structured JSON outputs, suitable for integration with human or AI workflows. Importantly, the model is designed for data abstraction rather than clinical diagnosis. Diagnostic interpretation, clinical reasoning, and final disease classification are performed by the reporting pathologist before the report is processed by the model. This study demonstrates the potential of using a fine-tuned LLM for clinical deployment, supporting expanded keratinocyte cancer registries and enabling more robust research into these common skin cancers.

## Supporting information

S1 Fig Training and evaluation loss finetuning Llama3.1-8B-Instruct model. Epoch: 3; leaning-rate: 2e-5; logging steps: 15; evaluation steps: 250. (S1A) Diagnosis only model. (S1B) Site only model.
(DOCX)

S1 Table F1-score and Kappa including 95% confidence interval for each lesion diagnosis from the diagnosis only model. N is the frequency of each diagnosis in the training dataset.
(DOCX)

S2 Table F1-score and Kappa including 95% confidence interval for each lesion site from the site only model. When the lesion site is “face”, the specific location on the face is extracted as well. N is the frequency of each site in the training dataset.
(DOCX)

S2 Fig Use of resources for finetuning the different models. (S2A) GPU memory use for the combined model. (S2B) GPU use for the combined model. (S2C) GPU memory use for the diagnosis model. (S2D) GPU for diagnosis model. (S2E) GPU memory use for the site model. (S2F) GPU for site model.
(DOCX)

S3 Table Lesion count for each pathology report for the QSkin-llama-3.1-8b answer and the manual review in STAR cohort text and pdf for all 5 runs. (DOCX)

S4 Table F1-score for diagnoses occuring ≤ 10 in the test set, predicted by QSkin-llama-3.1-8b (LLM) and the QSkin-ML (ML) model. *95% confidence intervals reflect variability across repeated LLM runs.
(DOCX)

S5 Table F1-score for lesion site occuring ≤ 10 in the test set, predicted by QSkin-llama-3.1-8b (LLM) and the QSkin-ML (ML) model. *95% confidence intervals reflect variability across repeated LLM runs.
(DOCX)
