# Supplementary figures and images for: Automated identification of keratinocyte cancers in pathology reports using large language models
Source: PLOS Digit Health. 2026 Jul 9;5(7):e0001547. doi: 10.1371/journal.pdig.0001547 (PMC13349157; doi:10.1371/journal.pdig.0001547)

S1A
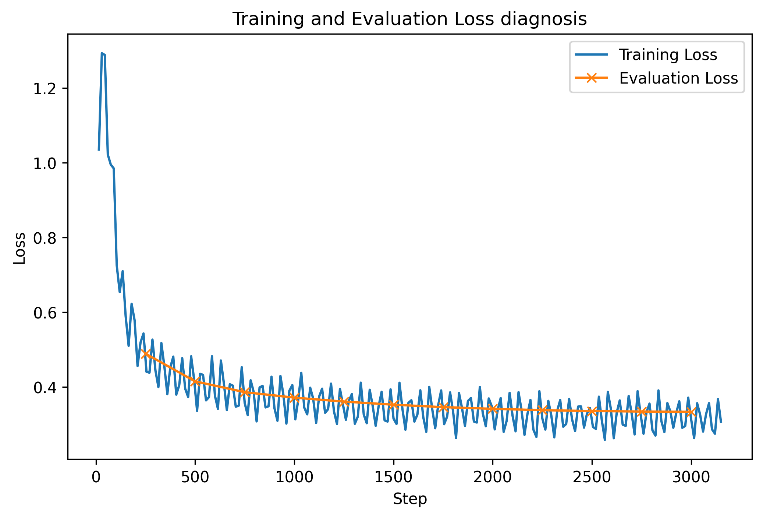


S1B
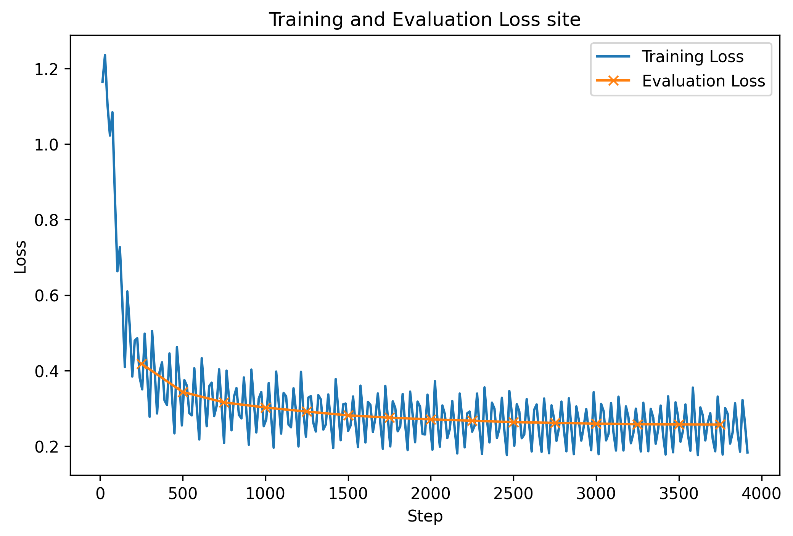

Supplement: S1 Fig — Epoch: 3; leaning-rate: 2e-5; logging steps: 15; evaluation steps: 250. (S1A) Diagnosis only model. (S1B) Site only model. (DOCX) [file pdig.0001547.s001.docx]

A
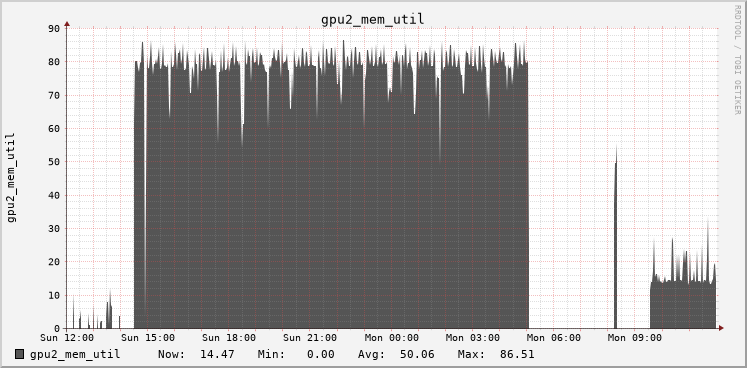


B
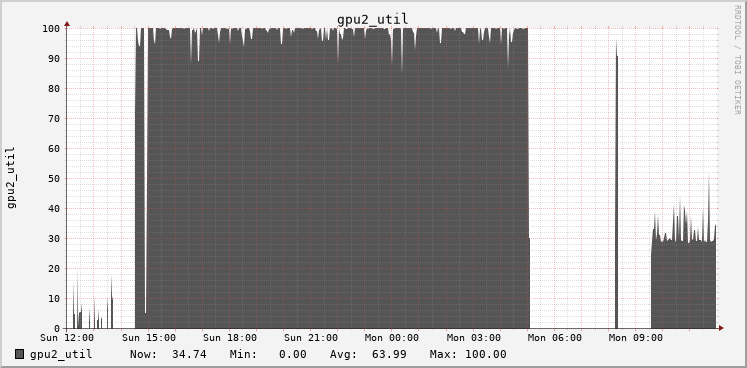


C
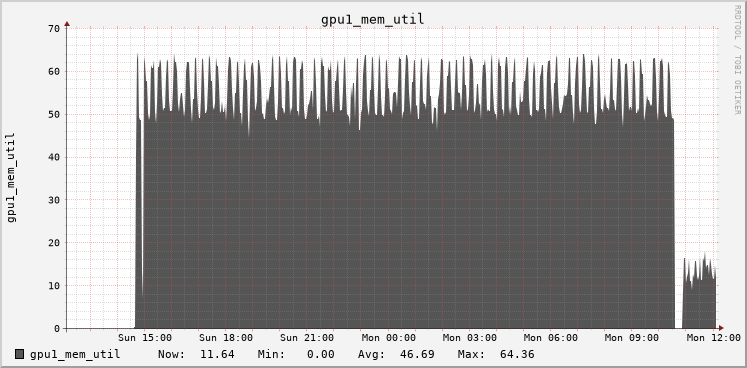


D
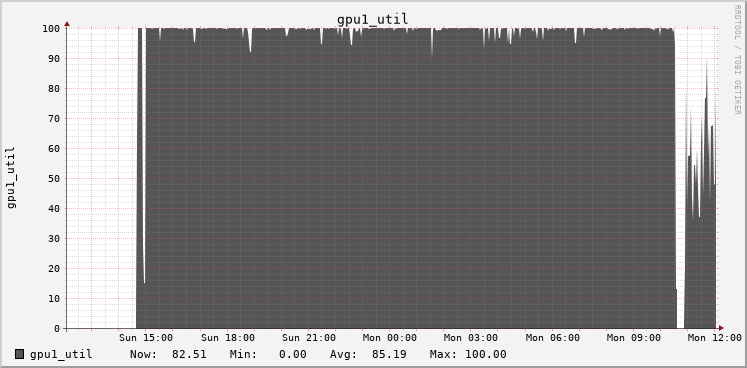


E
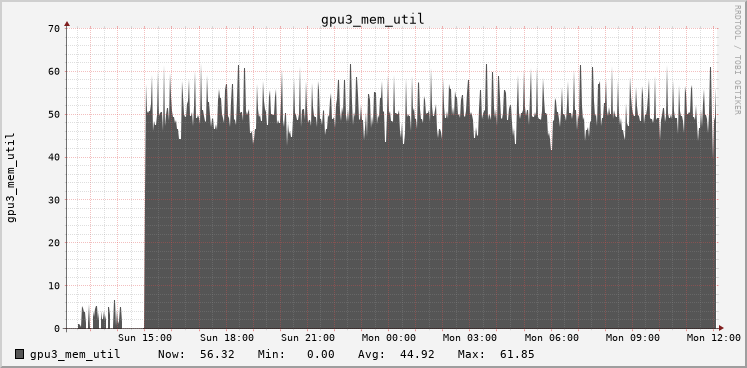


F
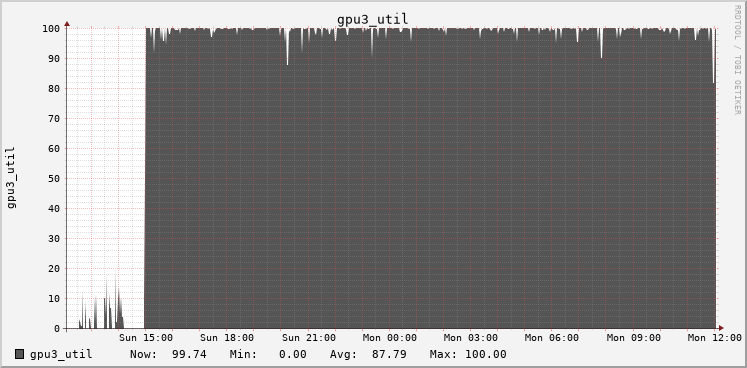

Supplement: S2 Fig — (S2A) GPU memory use for the combined model. (S2B) GPU use for the combined model. (S2C) GPU memory use for the diagnosis model. (S2D) GPU for diagnosis model. (S2E) GPU memory use for the site model. (S2F) GPU for site model. (DOCX) [file pdig.0001547.s004.docx]
