# Supplementary material for: Automated identification of keratinocyte cancers in pathology reports using large language models
Source: PLOS Digit Health. 2026 Jul 9;5(7):e0001547. doi: 10.1371/journal.pdig.0001547 (PMC13349157; doi:10.1371/journal.pdig.0001547)
Supplement: S1 Table — N is the frequency of each diagnosis in the training dataset. (DOCX) [file pdig.0001547.s002.docx]

| **Diagnosis** | **F1-score** | **Kappa** | **N training** |
| --- | --- | --- | --- |
| BCC | 0.92 [0.92, 0.92] | 0.89 [0.89, 0.90] | 8,860 |
| melanoma re-excision - clear | 0.88 [0.85, 0.90] | 0.87 [0.85, 0.90] | 413 |
| melanoma | 0.75 [0.72, 0.78] | 0.75 [0.72, 0.77] | 498 |
| intraepidermal carcinoma (IEC) | 0.90 [0.89, 0.90] | 0.87 [0.87, 0.88] | 6,085 |
| SCC | 0.91 [0.90, 0.91] | 0.90 [0.89, 0.90] | 3,107 |
| keratoacanthoma | 0.95 [0.95, 0.96] | 0.95 [0.94, 0.95] | 790 |
| dysplastic naevus | 0.19 [0.09, 0.30] | 0.19 [0.09, 0.30] | 139 |
| BCC re-excision - clear | 0.76 [0.75, 0.77] | 0.76 [0.74, 0.77] | 450 |
| solar keratosis | 0.74 [0.74, 0.74] | 0.71 [0.70, 0.71] | 4,682 |
| other | 0.03 [0.01, 0.05] | 0.02 [0.0, 0.04] | 319 |
| seborrhoeic keratosis | 0 | 0 | 250 |
| SCC re-excision - clear | 0.56 [0.51, 0.60] | 0.55 [0.51, 0.60] | 241 |
| lentigo maligna | 0.29 [0.19, 0.40] | 0.29 [0.18, 0.40] | 158 |
| squamo-proliferative lesions | 0.58 [0.53, 0.63] | 0.58 [0.53, 0.63] | 161 |
| IEC re-excision - clear | 0.62 [0.59, 0.64] | 0.61 [0.59, 0.64] | 169 |
| non-malignant lesion | 0.80 [0.79, 0.80] | 0.75 [0.74, 0.76] | 5,712 |

Excluded diagnoses with an occurrence of ≤ 10 in the text test set: *benign naevus*, *lentigo/solar lentigo*, and *no skin lesions*.
