# Supplementary material for: Automated identification of keratinocyte cancers in pathology reports using large language models
Source: PLOS Digit Health. 2026 Jul 9;5(7):e0001547. doi: 10.1371/journal.pdig.0001547 (PMC13349157; doi:10.1371/journal.pdig.0001547)
Supplement: S2 Table — When the lesion site is “face”, the specific location on the face is extracted as well. N is the frequency of each site in the training dataset. (DOCX) [file pdig.0001547.s003.docx]

| **Site** | **F1-score** | **Kappa** | **N training** |
| --- | --- | --- | --- |
| Abdomen | 0.73 [0.71, 0.76] | 0.73 [0.71, 0.76] | 226 |
| Back of hand | 0.84 [0.82, 0.85] | 0.83 [0.82, 0.84] | 1,477 |
| Back | 0.62 [0.60, 0.64] | 0.61 [0.59, 0.63] | 864 |
| Breast | 0.47 [0.45, 0.49] | 0.47 [0.44, 0.49] | 338 |
| Ears | 0.86 [0.86, 0.86] | 0.86 [0.85, 0.86] | 1,044 |
| Forearm, elbow, wrist | 0.87 [0.86, 0.87] | 0.85 [0.85, 0.85] | 3,345 |
| Lower back | 0.67 [0.66, 0.68] | 0.67 [0.66, 0.68] | 617 |
| Lower leg, ankle, knee | 0.91 [0.91, 0.92] | 0.90 [0.90, 0.90] | 4,150 |
| Neck | 0.85 [0.85, 0.86] | 0.84 [0.84, 0.85] | 1,452 |
| Non-skin | 0.90 [0.88, 0.92] | 0.90 [0.88, 0.92] | 1,421 |
| Palmer skin, fingers | 0.78 [0.75, 0.80] | 0.77 [0.75, 0.80] | 389 |
| Scalp | 0.79 [0.79, 0.80] | 0.79 [0.78, 0.80] | 861 |
| Shoulders | 0.80 [0.79, 0.81] | 0.79 [0.77, 0.80] | 1,823 |
| Thigh | 0.91 [0.90, 0.92] | 0.91 [0.90, 0.92] | 833 |
| Top of feet | 0.81 [0.78, 0.84] | 0.81 [0.78, 0.84] | 231 |
| Upper back | 0.68 [0.67, 0.69] | 0.66 [0.65, 0.67] | 1,629 |
| Upper chest/sternoclavicular | 0.73 [0.72, 0.73] | 0.71 [0.70, 0.72] | 1,396 |
| Upper arm | 0.85 [0.83, 0.86] | 0.84 [0.82, 0.85] | 1,650 |
| Face | 0.87 [0.87, 0.88] | 0.84 [0.83, 0.84] | 7,913 |
| Cheeks | 0.76 [0.76, 0.77] | 0.75 [0.74, 0.76] | 1,896 |
| Chin/jaw | 0.66 [0.64, 0.67] | 0.65 [0.63, 0.67] | 449 |
| Forehead | 0.79 [0.77, 0.80] | 0.78 [0.77, 0.80] | 1,343 |
| Lips | 0.83 [0.80, 0.86] | 0.83 [0.80, 0.85] | 473 |
| Nose | 0.89 [0.89, 0.90] | 0.89 [0.88, 0.89] | 1,878 |
| Skin of orbit/eyelid | 0.76 [0.75, 0.76] | 0.75 [0.74, 0.76] | 793 |
| Temple | 0.81 [0.79, 0.83] | 0.80 [0.78, 0.82] | 807 |

Excluded site with an occurrence of ≤ 10 in the text test set: *buttock*, *hip*, *no record*, and *plantar skin, toes.*
