# Supplementary material for: Automated identification of keratinocyte cancers in pathology reports using large language models
Source: PLOS Digit Health. 2026 Jul 9;5(7):e0001547. doi: 10.1371/journal.pdig.0001547 (PMC13349157; doi:10.1371/journal.pdig.0001547)
Supplement: S3 Table — (DOCX) [file pdig.0001547.s007.docx]

|  | Diagnosis count by QSkin-llama-3.1-8b | | | | | | | | | |  | |  | |  | |  | | | |
| --- | --- | --- | --- | --- | --- | --- | --- | --- | --- | --- | --- | --- | --- | --- | --- | --- | --- | --- | --- | --- |
| Diagnosis count by manually reviewed gold standard | **NaN** | **1** | **2** | **3** | **4** | **5** | **6** | **7** | **8** | **9** | | **10** | | **11** | | **12** | | **13** | **17** | **18** |
| **1** | 5 | 420 | 21 | 2 | 2 | 0 | 0 | 0 | 0 | 0 | | 0 | | 0 | | 0 | | 0 | 0 | 0 |
| **2** | 2 | 2 | 196 | 5 | 0 | 0 | 0 | 0 | 0 | 0 | | 0 | | 0 | | 0 | | 0 | 0 | 0 |
| **3** | 0 | 5 | 1 | 146 | 1 | 2 | 0 | 0 | 0 | 0 | | 0 | | 0 | | 0 | | 0 | 0 | 0 |
| **4** | 0 | 0 | 0 | 1 | 64 | 0 | 0 | 0 | 0 | 0 | | 0 | | 0 | | 0 | | 0 | 0 | 0 |
| **5** | 0 | 0 | 0 | 0 | 0 | 48 | 10 | 1 | 0 | 1 | | 0 | | 0 | | 0 | | 0 | 0 | 0 |
| **6** | 0 | 1 | 0 | 0 | 0 | 0 | 39 | 0 | 0 | 0 | | 0 | | 0 | | 0 | | 0 | 0 | 0 |
| **7** | 0 | 0 | 0 | 0 | 0 | 1 | 3 | 30 | 1 | 0 | | 0 | | 0 | | 0 | | 0 | 0 | 0 |
| **8** | 1 | 0 | 0 | 0 | 0 | 0 | 0 | 2 | 17 | 0 | | 0 | | 0 | | 0 | | 0 | 0 | 0 |
| **9** | 0 | 0 | 0 | 0 | 0 | 0 | 0 | 0 | 2 | 22 | | 1 | | 0 | | 0 | | 0 | 0 | 0 |
| **10** | 0 | 0 | 0 | 0 | 0 | 0 | 0 | 0 | 0 | 0 | | 5 | | 0 | | 0 | | 0 | 0 | 0 |
| **11** | 0 | 0 | 0 | 0 | 0 | 0 | 0 | 0 | 0 | 0 | | 0 | | 10 | | 0 | | 0 | 0 | 0 |
| **12** | 0 | 0 | 0 | 0 | 0 | 0 | 0 | 0 | 0 | 0 | | 0 | | 0 | | 0 | | 0 | 0 | 0 |
| **13** | 0 | 0 | 0 | 0 | 0 | 0 | 0 | 0 | 0 | 0 | | 0 | | 0 | | 0 | | 5 | 0 | 0 |
| **17** | 0 | 0 | 0 | 0 | 0 | 0 | 0 | 0 | 0 | 0 | | 0 | | 0 | | 1 | | 0 | 4 | 0 |
| **18** | 2* | 0 | 0 | 0 | 0 | 0 | 0 | 0 | 0 | 0 | | 0 | | 0 | | 0 | | 0 | 0 | 3 |

*Only lesion counts within the JSON structure {} were counted. LLM answer did not had a closing bracket, therefore, nothing was counted. After manual check: 17 lesions were found by LLM.
