# Supplementary material for: Automated identification of keratinocyte cancers in pathology reports using large language models
Source: PLOS Digit Health. 2026 Jul 9;5(7):e0001547. doi: 10.1371/journal.pdig.0001547 (PMC13349157; doi:10.1371/journal.pdig.0001547)
Supplement: S4 Table — *95% confidence intervals reflect variability across repeated LLM runs. (DOCX) [file pdig.0001547.s005.docx]

| **Diagnosis** | **F1-score LLM** | **F1-score ML** |
| --- | --- | --- |
| BCC | 0.95 [0.95, 0.95] | 0.93 |
| melanoma re-excision - clear | 0.88 [0.85, 0.90] | 0.88 |
| melanoma | 0.75 [0.72, 0.78] | 0.85 |
| intraepidermal carcinoma (IEC) | 0.90 [0.89, 0.90] | 0.86 |
| SCC | 0.91 [0.90, 0.91] | 0.91 |
| keratoacanthoma | 0.95 [0.95, 0.96] | 0.89 |
| dysplastic naevus | 0.19 [0.09, 0.30] | / |
| BCC re-excision - clear | 0.72 [0.70, 0.74] | 0.75 |
| solar keratosis | 0.76 [0.75, 0.77] | 0.75 |
| other | 0.03 [0.0, 0.06] | / |
| seborrhoeic keratosis | 0.16 [0.13, 0.19] | / |
| SCC re-excision - clear | 0.66 [0.61, 0.71] | 0.57 |
| lentigo maligna | 0.29 [0.21, 0.38] | / |
| squamo-proliferative lesions | 0.43 [0.39, 0.47] | 0.54 |
| iec re-excision - clear | 0.55 [0.46, 0.64] | 0.48 |
| non-malignant lesion | 0.84 [0.83, 0.84] | 0.91 |
