# Supplementary material for: Automated identification of keratinocyte cancers in pathology reports using large language models
Source: PLOS Digit Health. 2026 Jul 9;5(7):e0001547. doi: 10.1371/journal.pdig.0001547 (PMC13349157; doi:10.1371/journal.pdig.0001547)
Supplement: S5 Table — *95% confidence intervals reflect variability across repeated LLM runs. (DOCX) [file pdig.0001547.s006.docx]

| **Site** | **F1-score LLM** | **F1-score MK** |
| --- | --- | --- |
| Scalp | 0.81 [0.80, 0.82] | 0.87 |
| Ears | 0.89 [0.89, 0.90] | 0.88 |
| Neck | 0.91 [0.91, 0.91] | 0.88 |
| Shoulders | 0.81 [0.81, 0.81] | 0.81 |
| Upper chest | 0.72 [0.71, 0.73] | 0.76 |
| Breast | 0.29 [0.27, 0.32] | 0.56 |
| Abdomen | 0.88 [0.81, 0.94] | 0.84 |
| Back | 0.60 [0.59, 0.62] | 0.60 |
| Upper arm | 0.86 [0.85, 0.87] | 0.85 |
| Forearm, elbow, wrist | 0.86 [0.86, 0.86] | 0.92 |
| Back of hand | 0.86 [0.85, 0.86] | 0.90 |
| Palmar skin, fingers | 0.60 [0.58, 0.63] | 0.79 |
| Thigh | 0.89 [0.89, 0.90] | 0.91 |
| Lower leg, ankle, knee | 0.92 [0.91, 0.92] | 0.93 |
| Top feet | 0.87 [0.85, 0.88] | 0.90 |
| Upper back | 0.67 [0.66, 0.68] | 0.71 |
| Lower back | 0.52 [0.49, 0.56] | 0.63 |
| Face | 0.90 [0.90, 0.90] | 0.65 |
| Cheeks | 0.76 [0.75, 0.77] | 0.81 |
| Chin/jaw | 0.71 [0.71, 0.72] | 0.61 |
| Forehead | 0.81 [0.79, 0.82] | 0.82 |
| Lips | 0.86 [0.85, 0.87] | 0.88 |
| Nose | 0.89 [0.88, 0.89] | 0.92 |
| Skin of orbit/eyelid | 0.69 [0.69, 0.70] | 0.76 |
| Temple | 0.74 [0.72, 0.76] | 0.83 |
| Non-skin | 0.89 [0.88, 0.90] | 0.91 |
